# Safety and effectiveness of low-dose amikacin in nontuberculous mycobacterial pulmonary disease treated in Toronto, Canada

**DOI:** 10.1186/s40360-019-0302-1

**Published:** 2019-06-03

**Authors:** Maria Luisa Aznar, Theodore K. Marras, Ahmed Said Elshal, Mahtab Mehrabi, Sarah K. Brode

**Affiliations:** 1 Joint Division of Respirology, Department of Medicine, University Health Network and Sinai Health System, 399 Bathurst Street, Toronto, M5T 2S8 ON Canada; 2 Medicine Department, Vall d’Hebron University Hospital, PROSICS Barcelona, Universitat Autònoma de Barcelona, Barcelona, Spain; 3 Gastroenterology Department, National Hepatology and Tropical Medicine Institute, Cairo, Egypt; 4 West Park Healthcare Centre, Toronto, Canada

**Keywords:** Amikacin, NTM lung disease, Nontuberculous mycobacteria

## Abstract

**Background:**

Treatment guidelines suggest either a low-dose or high-dose approach when prescribing amikacin for nontuberculous mycobacterial pulmonary disease (NTM PD), but data supporting the low-dose approach are limited. The purpose of this study was to describe the safety and efficacy of the use of a low-dose of intravenous amikacin in a cohort of patients with NTM PD.

**Methods:**

We retrospectively reviewed all patients with NTM PD who received amikacin at our institution between July 1, 2003 and February 28, 2017. Demographics, clinical, microbiological and radiological data, indication and dose of amikacin, and adverse drug effects were recorded.

**Results:**

A total of 107 patients received a regimen containing amikacin for a median (IQR) of 7 (4–11) months. Seventy (65.4%) were female and the mean age (SD) was 58.3 (14.9) years. Amikacin was started at a median dose of 9.9 (2.5) mg/kg/day. Ototoxicity was observed in 30/77 (39%) patients and it was related to female sex (OR 4.96, 95%CI 1.24–19.87), and total dose of amikacin per bodyweight (OR 1.62, 95%CI 1.08–2.43). Patients of East Asian ethnicity were less likely to develop ototoxicity (0.24, 95%CI 0.06–0.95). Out of 96 patients who received amikacin for more than 3 months, 65 (67.7%) experienced symptom improvement and 30/62 (49.2%) converted their sputum to culture negative within a year.

**Conclusions:**

Patients with NTM PD treated with low-dose intravenous amikacin frequently developed ototoxicity, which was associated with female sex, and total dose of amikacin per bodyweight. Physicians should carefully consider dose, treatment duration, and long term prognosis in balancing risks and benefits of intravenous amikacin in NTM PD.

## Background

Nontuberculous mycobacteria (NTM) are ubiquitous microorganisms that may cause human infections, mainly affecting the lungs. Nontuberculous mycobacteria pulmonary disease (NTM PD) is often severe and requires a long course of multidrug therapy [1]. Despite treatment, prognosis is poor, and mortality remains high [1–4].

Use of injectable aminoglycosides has been associated with higher rates of sputum conversion compared with multidrug regimens not containing aminoglycosides in *Mycobacterium avium* complex (MAC) [5] and *Mycobacterium abscessus* (*M. abscessus*) pulmonary disease [6]. Guidelines from the American Thoracic Society / Infectious Diseases Society of America (ATS/IDSA) recommend amikacin use as first line therapy against *M. abscessus* pulmonary disease, advanced (especially fibrocavitary) or previously treated MAC pulmonary disease, and as second line therapy against some other NTM infections [1]. The well described ototoxicity, vestibular toxicity and renal toxicity due to amikacin limit the use of this drug for prolonged periods of time. Moreover, the correlation between clinical outcomes and amikacin susceptibility testing break-points in patients with NTM PD are not firmly established*.* Data suggest that a high Minimum Inhibitory Concentration (MIC) for amikacin is associated with worse clinical outcomes in MAC-PD [7, 8], although formal recommendations have not yet been developed for MAC-PD. There are no data regarding the association between MIC for amikacin and clinical outcomes in *M. abscessus*-PD, although *M. abscessus* is considered susceptible to amikacin at a MIC ≤16 mcg/ml [9]. Furthermore, although treatment guidelines suggest either a low-dose or high-dose approach when prescribing amikacin for NTM PD, data supporting the low-dose approach are scarce. Despite of all these limitations, amikacin remains one of the most potent drugs for the treatment of NTM PD, and therefore, data on its risks and benefits are critical.

The objective of this study was to describe the safety and efficacy of the use of low-dose intravenous amikacin in a cohort of patients with NTM PD.

## Methods

All patients with NTM-PD treated in the Toronto Western Hospital NTM clinic between July 1, 2003 and February 28, 2017 were retrospectively reviewed. Inclusion criteria for the study were: a) diagnosis of NTM-PD defined according to the ATS/IDSA criteria [1], and b) treatment regimen that included intravenous amikacin for any period of time. The study was approved by the University Health Network Research Ethics Board. Baseline demographic, clinical, microbiological and radiological data were collected.

The predominant radiological pattern was classified as nodular bronchiectatic, fibrocavitary, consolidation, random nodules and unclassifiable based on computed tomography (CT) [10]. Indication, dose and duration of amikacin were also recorded.

### Amikacin administration and toxicity

A Peripherally Inserted Central Catheter (PICC) was inserted and the first dose of amikacin was administered at the hospital and, if no adverse reactions, the following doses were administered at an outpatient setting (infusion clinic or patient residence), generally administered during a 30-min infusion. Routine amikacin peak levels were performed 30 to 45 min after amikacin infusion every month, with the subsequent doses adjusted accordingly. Amikacin dosing followed the low-dose approach suggested in the ATS/IDSA guidelines; the starting dose was 8–10 mg/kg, typically given thrice weekly (but ranging from 2 to 7 days per week), with the dose adjusted to target a peak level of 20–25 mg/ml [1]. During our study period, the ATS/IDSA guidelines did not significantly change with respect to amikacin dosing recommendations. The 1997 guidelines did not provide dosing recommendations for MAC, but for *M. abscessus* they recommend either a low or higher dose approach, either once or twice a day [11].

All patients who received amikacin for any duration were reviewed for toxicities. Blood work including serum creatinine was routinely performed every month while on amikacin. Nephrotoxicity was defined as increase in the absolute serum creatinine level of > 20 umol/L from baseline or > 20% change in subsequent testing as in previous studies [12]. Audiograms during amikacin therapy (advised to be done routinely) were recorded and compared with audiograms performed before starting amikacin. Bilateral hearing was tested at 250, 500, 1000, 2000, 4000, and 8000 Hz. Patients whose hearing threshold level were greater than 40 dB before starting amikacin were considered as having disabling baseline hearing impairment [13]. Ototoxicity was defined as a > 20 dB neurosensory hearing loss from baseline in either ear at any single frequency [14], seen on at least two consecutive audiograms, unless only one audiogram was performed after amikacin initiation. Persistent ototoxicity was defined if neurosensory hearing loss was observed after discontinuation of amikacin. Subjective symptoms of ototoxicity were also recorded. Vestibular toxicity was defined as having occurred if the patient reported compatible symptoms during a clinic visit, ie vertigo, disequilibrium, or ataxia, +/− nausea/vomiting or physical examination suggestive of vestibulotoxicity.

### Sputum culture and drug susceptibility testing

Specimen culture was performed using the Bactec 460 TB system (Becton Dickinson Microbiology Systems, USA) until May 2000 and, thereafter, with the Bactec MGIT 960 system (Becton Dickinson Microbiology Systems). Specimens were processed and analyzed as described by Hanna et al. [15]. NTM cultures were speciated using DNA probes (AccuProbe, Gen-Probe, USA) for MAC and *Mycobacterium gordonae*, and by high-performance liquid chromatography for other species [16]. Drug Susceptibility Testing was performed using the CLSI-recommended method of broth microdilution, using commercially prepared microtiter dilution plates (Thermo Fisher, Cleveland, OH) [9]. Concentrations of amikacin tested ranged from ≤1 to 64 μg/ml. Isolates with a MIC ≤16 were considered susceptible, those with a MIC of 32 or 64 were considered intermediate and those with a MIC > 64 were considered resistant [8, 16]. Since amikacin Drug Susceptibility Testing requires special request, it was not always requested in the early study period because of a lack of data regarding clinical correlation.

### Clinical and microbiological outcomes

Criteria for clinical outcomes and culture conversion have been previously defined [17]. Characteristics of patients who converted their sputum were compared with those of patients who continued to have positive cultures after starting amikacin.

### Statistical analysis

Descriptive statistics are presented as number (percent) and median (Interquartile range, IQR) or mean (Standard deviation, SD) depending on variable normality. The normal distribution of quantitative variables was tested through the Shapiro–Wilk test. Categoric variables were compared using chi-square tests or Fisher’s exact tests and continuous variables were compared using t-tests or Mann-Whitney tests. Demographics, dose and duration of amikacin, repeated exposure to amikacin, baseline weight, baseline serum creatinine and baseline disabling hearing impairment were selected as potential risk factors of ototoxicity. Demographics, NTM species, CT pattern, dose and duration of amikacin, and surgical management were selected as potential prognostic variables for culture conversion. We developed two models using a multivariate logistic regression analysis including ototoxicity and culture conversion as dependent variables. Variables with a *p*-value < 0.2 in the univariate analysis, and variables considered a priori to be clinically important, were included as independent variables. Multicollinearity was explored using variance inflation factor (VIF). A two-sided *P* value of **0.05** was used as the cutoff for statistical significance. All analyses were performed using SPSS statistical software (IBM SPSS version 23, Armonk, N.Y.).

## Results

One hundred and seven patients were treated with a regimen containing intravenous amikacin. Median duration of amikacin (IQR) was 7 (4–11) months. Table 1 shows baseline characteristics of patients treated with amikacin. Forty-four (41.1%) patients had previously been treated for NTM PD and one patient had received amikacin previously at another institution. Amikacin was started at a median (IQR) dose of 9.5 (8.3–10.4) mg/kg/day, thrice weekly in 77 (72%) patients, 5 days weekly in 20 (18.7%) patients, daily in 5 (4.7%) patients, twice weekly in 4 (3.7%) patients, and every 48 h in 1 (0.9%) patient. Amikacin was prescribed in combination with a multidrug oral antibiotic regimen in all patients, and 33 (30.8%) patients underwent lung surgery (30 patients underwent lung resection, 1 patient underwent lung transplant, 1 patient underwent cardiopulmonary transplant and 1 patient underwent a Clagett window). Twenty-four patients (22.4%) received a second course of amikacin for a median (IQR) of 4 (3–10) months, including 9 (37.5%) patients who restarted all antibiotics due to an infection recurrence and 15 (62.5%) who never stopped the oral antibiotics and were re-started on amikacin due to worsening of their symptoms. Nine patients (8.4%) received a third course of amikacin for a median (IQR) of 26 (4.5–35) months. Median (IQR) total dose of amikacin was 50.5 g (27.3–106.7).

**Table 1:** Baseline characteristics of patients treated with amikacin

Baseline characteristics	*N* = 107 (%)
Sex, Female	70 (65.4)
Age (Years), Mean (SD)	58.3 (14.9)
Race	
Caucasian	72 (67.3)
South Asian	4 (3.7)
East Asian	28 (26.2)
African	3 (2.8)
Comorbidities	
COPD/Emphysema	19 (17.7)
Previous TB	13 (12.1)
Cystic fibrosis	6 (5.6)
Asthma	5 (4.7)
Autoimmune disorders	5 (4.7)
Alpha 1 antitrypsin deficiency	2 (1.9)
Sarcoidosis	2 (1.9)
ABPA	2 (1.9)
Allogenic stem cell transplant	2 (1.9)
HIV infection	1 (0.9)
Species	
MAC^a^	69 (64.5)
*M. abscessus*	21 (19.6)
*M. xenopi*	11 (10.3)
MAC and *M. xenopi*	4 (3.7)
*M. shimodei*	1 (0.9)
*M. fortuitum*	1 (0.9)
AFB smear positive ever	100 (93.5)
Aspergillus isolation	39 (36.4)
CT pattern	
Nodular bronchiectatic	55 (51.4)
Fibrocavitary	40 (37.4)
Random nodules	2 (1.9)
Consolidation	0 (0)
Unclassifiable	10 (9.3)
Cavity	66 (61.7)
Indication for treatment	
First line	39 (36.4)
Second line	68 (63.6)

*SD* Standard deviation, *COPD* Chronic Obstructive Pulmonary Disease, *TB* Tuberculosis, *SLE* Systemic Lupus Erythematous, *ABPA* Allergic Bronchopulmonary Aspergillosis, *HIV* Human Immunodeficiency Virus, *MAC Mycobacterium avium* complex, *AFB* Acid Fast Bacilli, *CT* Computed Tomography

^a^Five patients had Clarithromycin resistant MAC; MAC isolates were not identified to the species level prior to 2010, and so are reported as MAC herein

The first course of amikacin was discontinued in 34 (31.8%) patients due to an adverse effect (17 (50%) ototoxicity, 6 (17.6%) fatigue, 4 (11.8%) renal insufficiency, 5 (14.7%) generalized skin rash, 1 (2.9%) paresthesias, and 1 (2.9%) restless extremities), 4 (3.7%) patients discontinued treatment due to poor improvement of symptoms and 4 (3.7%) discontinued amikacin for other reasons (need to travel in 1 case, need to treat another infection in 1 case, and poor adherence in 2 cases). Ten (9.3%) patients died while on amikacin. Causes of death were NTM progression in 6 patients, postoperative complication after adjuvant lung resection in 2 patients, aspergillosis in 1 patient and cardiac arrest in 1 patient. Out of 24 patients who received a second course of amikacin, 10 (41.7%) stopped the amikacin due to an adverse effect (5 ototoxicity, 2 skin rash, 2 PICC infection and 1 vestibular toxicity), 1 stopped amikacin due to poor clinical response, and 2 stopped amikacin due to a need to treat other medical conditions. Out of 9 patients who received a third course of amikacin, 2 were still on amikacin at the end of the study, 1 completed the treatment course, 4 developed an adverse effect (acute renal insufficiency in 1 patient, ototoxicity in 1 patient, skin rash in 1 patient and fatigue in 1 patient), and 2 died due to NTM progression.

### Adverse effects

Four (3.7%) patients developed vestibular toxicity after starting amikacin. Blood work was available in 96 patients, and it showed elevated serum creatinine concentrations in 6 (6.2%) patients. Renal insufficiency was reversible in 5 patients after stopping amikacin and in 1 after decreasing the dose of amikacin. Seven (6.5%) patients experienced a PICC associated thrombosis, one of them was diagnosed of a factor V Leyden deficiency thereafter, and one patient developed a *Staphylococcus aureus* bacteremia. Five patients developed a skin rash that disappeared after stopping the amikacin. Six patients experienced severe fatigue while on amikacin that lead to discontinuation of treatment. Table 2 summarizes the adverse effects associated with amikacin.

**Table 2:** Adverse effects associated with amikacin

Adverse effects	N (%)
Ototoxicity	
Subjective symptoms	43/107 (40.2%)
Hearing impairment seen in audiograms	30/77 (39.0%)
Nephrotoxicity	6/96 (6.2%)
Vestibular toxicity	4/107 (3.7%)
PICC related complications	
Venous thromboembolism	7/107 (6.5%)
Bloodstream infection	1/107 (0.9%)
Fatigue	6/107 (5.6%)
Skin rash	5/107 (4.7%)
Paresthesias	1/107 (0.9%)
Restless extremities	1/107 (0.9%)

*PICC* Peripherally Inserted Central Catheter

### Ototoxicity

Forty-three (40.2%) patients developed subjective ototoxicity while on amikacin; 32 reported tinnitus, 7 reported subjective hearing loss and 4 reported both symptoms. Audiograms were available in 77 (72%) patients. Five (6.5%) patients only had one follow up audiogram. Overall, there was objective ototoxicity in 30 (39%) patients; 16 (53.3%) of these 30 patients reported subjective ototoxicity. Out of 34 patients with subjective ototoxicity who had an audiogram, 16 (47.1%) had evidence of objective ototoxicity. Ototoxicity appeared a median of 5.5 (3–11) months after the initiation of amikacin. Although high frequency hearing loss (>2000 Hz) was seen most often, 3 (10%) of the 30 patients showed deficits in both high and low frequencies.

Patients developing ototoxicity were more likely to be female (OR 4.96, 95%CI 1.24–19.87, *p* = 0.024), received a higher total dose of amikacin per baseline bodyweight (g/kg) (OR 1.62, 95%CI 1.08–2.43, *p* = 0.020) and were less likely to be of East Asian ethnicity (OR 0.24, 95%CI 0.06–0.95, *p* = 0.042) than patients who did not develop ototoxicity. This information is summarized in Table 3.

**Table 3:** Comparison of demographic and clinical characteristics in patients with and without audiographically demonstrated ototoxicity

	Ototoxicity *N* = 30	No ototoxicity *N* = 47	Univariate model		Multivariate model^a^	
			OR (95%CI)	P value	OR (95%CI)	P value
Sex, female	24 (80%)	29 (61.7%)	2.48 (0.85–7.24)	0.096	4.96 (1.24–19.87)	0.024
Age (years), median (IQR)	61 (43–65)	65 (51–72)	0.98 (0.95–1.01)	0.247	0.98 (0.94–1.02)	0.297
Race						
Caucasian	22 (73.3%)	29 (61.7%)	1.71 (0.63–4.64)	0.295		
South Asian	1 (3.3%)	1 (2.1%)	1.59 (0.09–26.36)	0.748		
East Asian	6 (20%)	16 (34%)	0.48 (0.16–1.42)	0.188	0.24 (0.06–0.95)	0.042
African	1 (3.3%)	1 (2.1)	1.59 (0.09–26.36)	0.748		
Weekly dose AK per bodyweight (mg/kg/week), median (IQR)^b^	29 (23.7–41.3)	25.2 (21.8–29.4)	1.05 (1.00–1.10)	0.047	1.03 (0.98–1.09)	0.248
Months on AK, median (IQR)	11.5 (7–18.25)	7 (4–10)	1.04 (1.00–1.08)	0.052		
Total dose AK per bodyweight (g/kg), Median (IQR)^c^	1.81 (0.85–3.13)	0.74 (0.51–1.27)	1.59 (1.08–2.33)	0.018	1.62 (1.08–2.43)	0.020
Total dose AK (g), Median (IQR)^d^	77.3 (52.9–196.3)	41.7 (25.6–67.5)	1.01 (1.00–1.02)	0.011		
Repeated exposure to amikacin	9 (30%)	10 (21.3%)	1.59 (0.56–1.52)	0.388		
Baseline weight (kg), Median (IQR)	51.5 (41–66.5)	55 (48–64)	0.99 (0.96–1.03)	0.712		
Initial serum creatinine (umol/L), mean (SD)	66.0 (56.7–74.0)	63 (53–75)	1.01 (0.97–1.04)	0.656		
Baseline disabling hearing impairment	8 (26.7%)	24 (51.1%)	0.35 (0.13–0.94)	0.037	0.54 (0.17–1.74)	0.305
Current treatment with macrolides	29 (96.7%)	45 (95.7%)	1.23 (0.11–14.87)	0.839		
Nephrotoxicity	1 (3.6%)	4 (8.5%)	0.38 (0.04–3.58)	0.398		

*IQR* Interquartile Range, *AK* Amikacin

^a^Variables with a p-value < 0.2 in the univariate analysis, and variables considered a priori to be clinically important, were included in the multivariate model, wherein multicollinearity was explored using variance inflation factor (VIF). Months on amikacin and total dose amikacin per bodyweight both had VIFs> 10, and so were eliminated

^b^OR represents risk associated with each increase of 1 mg/kg/week

^c^OR represents risk associated with each increase of 1 g/kg total dose

^d^OR represents risk associated with each increase of 1 g total dose

Total dose of amikacin and months on amikacin were excluded from the multivariate analysis due to collinearity

### Amikacin MIC

Twenty-six patients (14 infected by MAC and 12 infected by *M. abscessus*) had amikacin Drug Susceptibility Testing before initiating this agent. Out of 14 MAC isolates tested, 11 (78.6%) showed susceptibility to amikacin, 2 (14.3%) showed intermediate resistance and 1 (7.1%) was resistant to amikacin. Eleven (91.7%) *M abscessus* isolates were susceptible to amikacin, and 1 (8.3%) was resistant. Three out of 4 patients whose isolates were either intermediate or resistant to amikacin had not been previously exposed to aminoglycosides. Thirteen patients had repeat Drug Susceptibility Testing after receiving amikacin and one of them developed amikacin resistance.

### Microbiological and clinical outcomes

Sixty-five (67.7%) of the 96 patients who received a first course of amikacin for ≥3 months experienced improvement in their symptoms after starting amikacin and 31 (45.6%) out of 68 patients who had a previous positive sputum culture before starting amikacin and follow up sputum after amikacin initiation converted their sputum within a year following the initiation of amikacin. Twenty-six patients experienced persistent culture conversion. None of the 15 patients who received a recurrent (second or third) course of amikacin for ≥3 months and who had a positive culture before starting amikacin achieved sputum culture conversion.

Patients who achieved sputum culture conversion underwent surgery more often than those who did not achieve sputum culture conversion (61.3% vs 5.4%, *p* < 0.001). Excluding patients who underwent surgery, 12/47 (25.5%) of patients achieved sputum culture conversion. One of the 2 patients with a MIC for amikacin > 64 achieved sputum culture conversion. In the multivariate logistic regression model, surgical intervention was the only variable associated with sputum culture conversion (OR 33.01, 95%CI 4.79–22.70, p < 0.001). Factors associated with sputum culture conversion are shown in Table 4.

**Table 4:** Patient characteristics and their association with sputum culture conversion

	Sputum culture conversion *N* = 31	No sputum culture conversion *N* = 37	Univariate model		Multivariate model	
			OR (95%CI)	P value	OR (95%CI)	P value
Sex, female	23 (74.2%)	23 (62.2%)	1.75 (0.62–4.97)	0.293		
Age (years), median (IQR)	58 (50–67)	61 (44–47.5)	1.00 (0.97–1.03)	0.953		
Species/complex						
MAC	17 (54.8%)	20 (54.1%)	1.02 (0.38–2.73)	0.969		
*M. abscessus*	3 (9.7%)	10 (27%)	0.28 (0.76–22.23)	0.073	0.87 (0.18–4.28)	0.868
*M. xenopi*	6 (19.4%)	2 (5.4%)	4.12 (0.76–22.23)	0.099	9.50 (0.68–131.82)	0.094
MAC/*M. xenopi*	1 (3.2%)	2 (5.4%)				
Macrolide resistant MAC	2 (6.5%)	3 (8.1%)	0.76 (0.12–4.89)	0.774		
*M. simiae*	1 (3.2%)	0				
*M. fortuitum*	1 (3.2%)	0				
FC pattern	13 (35.1%)	13 (41.9%)	1.33 (0.50–3.56)	0.566		
Cavity	21 (67.7%)	23 (62.2%)	1.28 (0.47–3.49)	0.632		
AFB positive ever	30 (96.8%)	37 (100%)	3.69 (0.15–93.81)	0.791		
Total dose AK per bodyweight (g/kg), median (IQR)^b^	0.79 (0.55–2.29)	1.20 (0.74–2.09)	0.81 (0.55–1.18)	0.268	0.54 (0.26–1.10)	0.089
Weekly dose AK per bodyweight (mg/kg/week), median (IQR)^a^	26.09 (23.61–33.87)	27.03 (22.6–40.3)	0.98 (0.94–1.03)	0.438		
Months on AK, median (IQR)	8 (4–18)	8 (6–15)	0.98 (0.94–1.02)	0.356		
Total dose AK (g), Median (IQR)^c^	46.8 (29.4–130.3)	60.3 (37.8–118.0)	1.00 (0.99–1.01)	0.725		
Surgery	19 (61.3%)	2 (5.4%)	27.7 (5.61–136.9)	< 0.001	33.01 (4.79–227.27)	< 0.001

*FC* Fibrocavitary pattern, *IQR* Interquartile Range, *MAC Mycobacterium avium* Complex, *AK* Amikacin

^a^OR represents risk associated with each increase of 1 mg/kg/week

^b^OR represents risk associated with each increase of 1 g/kg total dose

^c^OR represents risk associated with each increase of 1 g total dose

## Discussion

We observed that ototoxicity was common, and it was associated with female sex, and total dose of amikacin per bodyweight in patients with NTM PD. Vestibular toxicity and nephrotoxicity were not frequently observed. Most patients had symptomatic improvement after starting amikacin, however, only half of them achieved sputum culture conversion.

Amikacin has been shown to be an effective drug in the management of NTM PD, especially in patients with cavitary disease and in those whose isolate is macrolide resistant MAC or *M. abscessus* [6, 18, 19]*.* However, the potential adverse effects due to aminoglycosides limit the use of these drugs. The most common adverse effects due to aminoglycosides are ototoxicity, vestibular toxicity and nephrotoxicity [20, 21]. Previous studies describing patients with NTM pulmonary disease treated with regimens containing aminoglycosides have reported a variable incidence of adverse effects, although doses and duration of amikacin varied among studies [6, 12, 14].

In our study, we observed that 39% of patients experienced ototoxicity after a median of 5.5 (3–11) months of amikacin. Peloquin and colleagues observed a similar incidence of ototoxicity (37%). Although the doses used in our study are lower than the doses used in the Peloquin study (median dose of 9.5 mg/kg thrice weekly vs. 15 mg/kg daily or 25 mg/kg thrice weekly, respectively), the total duration of amikacin used in our study was longer (median 33 vs. 15 weeks), so the total dose of amikacin per kilogram bodyweight was similar (909 mg/kg in our study vs. 1012 mg/kg in the daily dose group and 967 mg/kg in the thrice-weekly dosing group in the Peloquin study). Lee et al. observed a 25% rate of ototoxicity in patients infected by *M. abscessus* receiving a median dose of amikacin of 19.3 mg/kg/day during 4 weeks [22]. Ellender et al. observed a 18% rate of ototoxicity in patients with NTM PD receiving a median dose of 22 mg/kg/day for a median of 8.64 weeks [6, 12]. Although the total dose of amikacin per bodyweight was not provided from the studies by Ellender et al. and Lee et al., it can be calculated that patients received approximately 462 mg/kg and 540 mg/kg cumulative doses respectively; the lower total dose per bodyweight used in these studies may explain the lower rates of ototoxicity observed, compared with the Peloquin study and our study.

We observed that most patients with ototoxicity experienced hearing impairment in higher frequencies. Aminoglycosides cause degeneration of hair cells in the organ of Corti, predominantly in the basal turn which is required to sense high-frequency sounds, so high-frequency hearing loss is more common in patients developing ototoxicity related with aminoglycosides [23]. Half of our patients with subjective symptoms of ototoxicity had a normal audiogram. The absence of objective hearing changes in patients reporting symptoms of ototoxicity have been previously reported [14] and it might be due to different mechanisms of ototoxicity. Some of the subjective symptoms could be related to an acute and not permanent toxicity due to high dose aminoglycosides and may not be reflected in audiograms [24]. In addition, we did not review changes in speech recognition thresholds, which may potentially vary from changes in the pure-tone audiogram thresholds.

Different factors have been associated with an increased risk of ototoxicity. Age, male sex, low body weight, total dose of amikacin and duration of amikacin have been reported as risk factors for ototoxicity and nephrotoxicity [14, 25–29]. In our study, we observed that females and patients who received a higher total dose of amikacin per bodyweight were more likely to experience ototoxicity. We also observed that East Asian patients were less likely to develop ototoxicity than patients from other ethnicities. The explanation of this finding is not clear. Although some mitochondrial mutations have been linked to ototoxic responses to aminoglycosides, it is not clear if there are differences in the prevalence of these mutations along ethnic groups [30]. Macrolides have been also associated with ototoxicity [31, 32]. We did not observe differences regarding macrolide use between patients who developed ototoxicity and those who did not, but since almost all of our patients received macrolides, our dataset lacked adequate variability to address this question.

Vestibular toxicity observed in our study was 3.7%, lower than in previous reports (7.4–9%) [14, 33]. However, our data were based on patient self-reported symptoms rather than physical examination focused on vestibular toxicity, so we may have missed some cases of toxicity. Six patients experienced nephrotoxicity, and serum creatinine returned to normal values in all of them after either stopping or decreasing the dose of amikacin. Nephrotoxicity is another concerning adverse effect associated with the use of aminoglycosides. However, it is more often observed in patients receiving several doses a day and those who already have baseline renal impairment [34]. Previous series reporting patients with NTM PD treated with intravenous amikacin have not observed nephrotoxicity as a common adverse effect [35]. We also observed that 6 patients developed a PICC associated thrombosis and one patient developed a PICC associated systemic infection, similar to rates previously reported [36].

Twenty-six patients had amikacin Drug Susceptibility Testing performed before starting this agent and two of them (1 MAC and 1 *M. abscessus*) had an amikacin MIC >64. Although correlation between break points and clinical outcomes are not clear, it seems that patients infected by MAC whose isolates have a MIC for amikacin > 64 have a poor prognosis compared with those who have lower MICs [7, 8]. In our study, one of the two patients with an isolate with a MIC >64 achieved sputum conversion. Interestingly, neither of these patients was known to have previously received aminoglycosides.

Symptomatic improvement after taking amikacin was observed in 68% of patients, and persistent sputum culture conversion within a year after the initiation of amikacin was observed in 46% of patients (25% treated exclusively with antibiotics). Ellender at al. had similar results; 76% of patients achieved a symptomatic improvement and 38% achieved persistent sputum culture conversion [12]. Although the proportion achieving sputum culture conversion was somewhat higher in our study, 21 (20%) of our patients underwent surgical resection, compared with no patients in their study. Namkoong et al. presented a small number of patients with *M. abscessus* pulmonary disease, and 8/13 (61.5%) achieved persistent sputum culture conversion; they also used a higher daily dose and shorter duration of amikacin than in our study, but did not report total dose [6]. Other studies describing amikacin administration protocols and adverse events in NTM-PD did not present data on clinical outcomes [14, 37]. Given the limited studies describing amikacin use in NTM-PD, and the heterogeneity in the patients studied in terms of NTM species treated, severity of disease, companion drugs, and use of surgery, it is difficult to draw conclusions regarding the optimal amikacin protocol to maximize clinical outcomes and minimize toxicity. The ATS/IDSA guidelines describe either low-dose or high-dose approaches, noting that high-dose is not likely to be tolerated for long durations that may be required to control NTM-PD. It is clear that the cumulative dose of amikacin is reliably associated with ototoxicity. In our clinical experience the large majority of NTM-PD patients prefer to withhold amikacin (and experience worsening NTM-PD) rather than experience severe hearing impairment. We think it is important to consider both of the above when crafting a regimen for individual patients. In situations where definitive therapy is possible, like surgical resection of limited disease, an intensive high-dose, short-term regimen may be preferable, assuming the likelihood of recurrence is low. In situations where disease suppression is the goal, where it is likely that amikacin will be useful for a prolonged period or in recurrent courses, then a low-dose regimen may be preferred.

We also observed that none of the patients who received a subsequent course of amikacin achieved sputum culture conversion. Although no previous studies have reported specific data about subsequent courses of amikacin, patients previously treated for NTM lung disease are less likely to achieve sputum culture conversion than patients not previously treated [38]. We observed that patients who underwent lung resection for the management of NTM pulmonary disease were more likely to achieve sputum culture conversion than patients treated exclusively with antibiotics. This finding has been previously reported for patients with an infection caused by *M. abscessus* and macrolide resistant MAC [4, 19].

Our study has several limitations. First, some of the adverse effects attributed to amikacin may have been due to another of the multiple accompanying antibiotics that patients received for the treatment of NTM-PD. However, the adverse effects reported in the study were thought to be related to amikacin. Second, the study was conducted retrospectively, so information was not collected in a standardized fashion. This is particularly relevant in that not all patients had audiograms performed. Although we recommend baseline and routine follow-up audiometry for all amikacin-treated patients, patients with more risk factors or subjective symptoms of ototoxicity may have been more adherent to this recommendation. Accordingly, the risk of ototoxicity may be overestimated. Third, there may have been differences in management decisions during the long time period of our study. However, as noted above, the ATS/IDSA guidelines did not significantly change with respect to amikacin dosing recommendations during our study period. Likewise our approach did not significantly change, typically following a lower dose strategy because of our patients’ age range and strong aversion to the risk of ototoxicity as well as the high rates of recurrence of NTM lung disease with the potential need for retreatment with amikacin.

## Conclusions

We observed that patients with NTM-PD treated with low-dose intravenous amikacin frequently developed ototoxicity, and it was associated with female sex, and total dose of amikacin per bodyweight. The symptoms of most patients improved after starting amikacin, and half of them achieved sputum culture conversion. More research is needed regarding the role of amikacin in NTM-PD, and the optimal protocol to achieve the most benefit with the least adverse effects.

## Abbreviations

ATS/IDSA: American Thoracic Society / Infectious Diseases Society of America
CT: Computed tomography
dB: Decibels
IQR: Interquartile range
MAC: *Mycobacterium avium* complex
MIC: Minimum Inhibitory Concentration
NTM PD: NTM pulmonary disease
NTM: Nontuberculous mycobacterial
PICC: Peripherally Inserted Central Catheter
SD: Standard deviation
VIF: Variance inflation factor
